# Gut microbiota changes require vagus nerve integrity to promote depressive-like behaviors in mice

**DOI:** 10.1038/s41380-023-02071-6

**Published:** 2023-05-02

**Authors:** Eleni Siopi, Mathieu Galerne, Manon Rivagorda, Soham Saha, Carine Moigneu, Stéphanie Moriceau, Mathilde Bigot, Franck Oury, Pierre-Marie Lledo

**Affiliations:** 1 Institut Pasteur, Université Paris Cité, CNRS UMR 3571, Perception and Memory Unit, 75015 Paris, France; 2 Université Paris Cité, CNRS, INSERM, Institut Necker Enfants Malades-INEM, 75015 Paris, France; 3 Platform for Neurobehavior and Metabolism, Structure Fédérative de Recherche Necker, 26 INSERM US24/CNRS UAR 3633, 75015 Paris, France

**Keywords:** Biological sciences, Neuroscience

## Abstract

Chronic stress constitutes a major risk factor for depression that can disrupt various aspects of homeostasis, including the gut microbiome (GM). We have recently shown that GM imbalance affects adult hippocampal (HPC) neurogenesis and induces depression-like behaviors, with the exact mechanisms being under active investigation. Here we hypothesized that the vagus nerve (VN), a key bidirectional route of communication between the gut and the brain, could relay the effects of stress-induced GM changes on HPC plasticity and behavior. We used fecal samples derived from mice that sustained unpredictable chronic mild stress (UCMS) to inoculate healthy mice and assess standard behavioral readouts for anxiety- and depressive-like behavior, conduct histological and molecular analyses for adult HPC neurogenesis and evaluate neurotransmission pathways and neuroinflammation. To study the potential role of the VN in mediating the effects of GM changes on brain functions and behavior, we used mice that sustained subdiaphragmatic vagotomy (Vx) prior the GM transfer. We found that inoculation of healthy mice with GM from UCMS mice activates the VN and induces early and sustained changes in both serotonin and dopamine neurotransmission pathways in the brainstem and HPC. These changes are associated with prompt and persistent deficits in adult HPC neurogenesis and induce early and sustained neuroinflammatory responses in the HPC. Remarkably, Vx abrogates adult HPC neurogenesis deficits, neuroinflammation and depressive-like behavior, suggesting that vagal afferent pathways are necessary to drive GM-mediated effects on the brain.

## Introduction

Mounting data show that the gut microbiome (GM) can influence plasticity in the hippocampus (HPC) and impact affective behaviors, yet the precise underlying mechanisms are still poorly documented [1–4]. The gastrointestinal branches of the vagus nerve (VN), which constitutes a direct bidirectional route of communication between the gut and the brain, are well-suited to carry neural messages associated with changes in peripheral states to the brain [5–11]. For instance, the ability of some gut bacterial strains to induce anxiety-like behaviors and alter the expression of brain derived neurotrophic factor (BDNF) and GABA receptor subunits in the HPC depends on vagal afferents [7–9]. Similarly, gut vagal sensory signaling affects HPC plasticity and adult neurogenesis [10, 11]. On one hand, impairments in HPC neurogenesis are associated with depressive states, and on the other hand, neurogenic stimuli such as fluoxetine or physical exercise, boost HPC neurogenesis and counteract depression [12–16].

Chronic stress is a major risk factor for the development of depression. It induces gut microbiota changes and HPC impairments in both mice and humans, which are due to some extent to deficits in adult HPC neurogenesis [3, 4, 17–22]. We have recently provided proof that chronic stress-related GM disturbances constitute a causal factor in the development of depressive states and adult HPC neurogenesis deficits in mice, by hijacking tryptophan metabolism and brain serotonin bioavailability [3]. Interestingly, VN afferents innervate brainstem nuclei that are vital for serotonin bioavailability in the brain, namely the serotonergic dorsal raphe nucleus which provides serotonergic innervation throughout the brain, including the HPC, and regulates HPC neuroplasticity [23–25]. Importantly, it has been reported that VN stimulation increases serotonergic input into HPC neurons and influences affective behaviors in rats [26]. Altogether, these observations raise the question of whether the GM harnesses the VN machinery to regulate key neurotransmission pathways in the brain and influences HPC neuroplasticity and behavior.

To address this question, we employed the unpredictable chronic mild stress (UCMS) model of induced depression-like behavior and investigated whether the ensuing GM perturbations require an intact VN to impact on adult HPC neurogenesis and promote depressive-like behavior.

## Materials and methods

### Animals

Experiments were performed using adult (8–10 weeks old) male C57BL/6j mice purchased from Janvier labs (St Berthevin, France). They were housed in groups of five and maintained in standard conditions (controlled room temperature and humidity, 12 h/12 h light/dark cycle, with lights on at 8:00 AM, ad libitum access to dry food pellets and water) at the Pasteur Institute animal care facility, officially registered for experimental studies on rodents (Approval number for animal care facility: A 75-15-01-6 2014-720). All studies were performed in compliance with the French legislation (Decree of 1 February 2013, 2013-118) and the European Communities Council Directive of 22 September 2010 (2010/63/EEC). All animal experiments were designed according to the 3 R’s rules and approved by the French Ministry of Research (projects CETEA #2013-0062, #2016-0023). Sample size was chosen using the freely downloadable G power software, to ensure adequate statistical power all the while respecting the 3 R principle. Behavioral analyses included 10 subjects per group, cellular analysis included 6 to 8 samples per group and molecular analyses included 5 samples per group.

### Unpredictable chronic mild stress (UCMS) procedure

Mice were chronically exposed to interchanging unpredictable mild stressors, administered daily in random order for 8 weeks, as previously described [3, 4, 27, 28]. Two stressors were applied daily, one in the morning and one in the evening, and none of the stressors involved food or water deprivation. The different stressors included cage shaking (5 min), cage tilting 45 °C (2 H), moist bedding (2 H), overnight illumination (12 H), reversing day and night (24 H), restraint (30 min), recurrent cage change (2 H) and exposure to predator odors (rat or fox urine). Behavioral experiments were performed over a period of 1 week, starting at week 8 of the UCMS protocol. At week 9, mice were euthanized and randomly allocated to histological or molecular analyses using a counter-balanced design (see Supplementary Table 1). The control animals were socially housed (5 animals/cage) and left undisturbed except for necessary procedures, including routine cleaning.

### Treatments

The antibiotic (ABX) compounds were applied in drinking water during one week and consisted of a mixture of ampicillin (1 mg/ml), streptomycin (5 mg/ml), colistin (1 mg/ml), vancomycin (0.5 mg/ml) and amphotericin (0.1 mg/ml), as previously described [3]. Overall, we did not witness any significant ABX-induced changes in stool consistency/shape, presence of mucus or blood or diarrhoea-induced fur staining. One day prior microbiota transplantation, the ABX treatment was discontinued and replaced by sterile water. The fecal samples used for the transfer protocol were harvested from donor mice (CT or UCMS) the day of the inoculation. The fecal suspension was produced by dissolving 1 mg of feces in 5 ml sterile PBS. The fecal suspension (300 μl per mouse) was delivered by oral gavage 1 and 4 days following ABX discontinuation.

### Subdiaphragmatic vagotomy

Subdiaphragmatic vagotomy was performed as previously described [7, 11]. Mice were anesthetized with ketamine/xylazine (10 mg/g BW, Sanofi). The stomach and lower esophagus were gently exposed following a mid-lateral incision of the skin and abdominal wall, and the intestine was retracted to allow access to the stomach. A ligature was placed around the esophagus at its entrance to the stomach to allow for gentle retraction and clearly expose both vagal trunks. These were dissected and all neural and connective tissue surrounding the esophagus below the diaphragm was removed to transect all small vagal branches. A 2-week recovery period was allowed before the behavioral experiments took place.

### Experimental sets

A total of 280 mice were used in this study, allocated in six separate experimental sets. All of the data presented herein have been reproduced at least twice in the host laboratory, with some results (for instance, impact of microbiota inoculation on behavior and neurogenesis) being replicated up to four times. All subjects euthanized at belated time-points (3 and 7 weeks post-inoculation) were subjected to behavioral analyses and were then assigned to cellular or molecular analyses by simple randomization. The latter was performed by creating a list of subjects and treatments using the Microsoft Excel’s function RAND. Analyses were performed by an investigator who was blind to the experimental conditions and used numerical coding. Sample size was determined by power analysis, using the G Power software. At least 10 animals per group were included in the behavioral analyses and 5–8 samples per group were included in the molecular and cellular analyses. A detailed schematic representation of all the experimental sets used in this study can be found in Supplementary Table 1.

### Behavioral assessment

For all behavioral tests, mice were transferred to the testing room at least 1 H before testing. GM donor mice were tested over a period of 1 week while the animals sustained the last week of UCMS. GM recipient mice were tested upon exit from the isolators. All analyses were performed with the experimenter blind to the experimental condition.

#### Open field

Animals were placed in white Plexiglas containers (43 × 43 cm) and their behavior was recorded by a video camera during 30 min. A tracking system (Noldus Ethovision 3.0) was used to map center and periphery zones and to calculate the time spent in each zone.

#### Elevated plus maze

The test was conducted using a plus-cross-shaped apparatus made of black Plexiglas that was elevated 58 cm above the floor and comprised two open and two closed arms (30 × 6 cm) that extended from a central platform (7 × 7 cm). The Noldus Ethovision 3.0 tracking system was used to record behavior for 6 min. The time spent and number of entries in the center, open arm, and closed arm zones were calculated.

#### Light and dark box

A two-compartment box containing a dark chamber (black walls with upper lid) and a light chamber (300 lux, white Plexiglas walls, no upper lid) was used. The chambers were connected by a 10 × 10 cm door in the middle of the wall. Animals were placed in one corner of the light chamber facing the wall and were allowed to freely explore for 10 min. The Noldus Ethovision 3.0 tracking system was used to record behavior. The number of entries and the total time spent in the light chamber were estimated as a proxy of anxiety-like states.

#### Sucrose preference test

Mice were given for 24 H a free choice between two bottles, one filled with a sucrose solution (1%, diluted in drinking water) and another one filled with drinking water. To avoid potential bias of side preference in drinking behavior, the position of the bottles was switched after 12 H. The consumption of water and sucrose solution was calculated by weighing the bottles. Sucrose preference was measured as the percentage of consumed sucrose solution to the total amount of solution consumed (sucrose and water).

#### Novelty suppressed feeding test

The test container used was a white plastic box (50 × 50 × 20 cm) whose floor was covered by wooden bedding. Twenty-four hours prior testing, all food was removed from the home cage. A single food pellet (regular chow) was adhered on a piece of Whatman paper and positioned in the center of the container that was brightly illuminated. The latency to eat was measured during 10 min. At the end of each session, the animals were transferred to their home cage and the amount of food consumed over the subsequent 5-min period was measured as a control of feeding drive.

#### Tail suspension test

Mice were suspended at approximately one-third from the end of the tail, using regular tape, to an aluminum bar connected to a strain gauge. Tape was affixed to the mouse’s tail 2 cm from the tip and the mouse was suspended from a metal rod at a height of 30 cm. The test was recorded during a 5-min period. Upon viewing of the video recordings, the total time spent in an immobile posture was measured. Mice were considered immobile when they stopped struggling to escape and hung passively, motionless, by the tail.

#### Forced swim test

Mice were placed into a clear Plexiglas cylinder (25 cm in height and 10 cm in diameter) filled with water (22 °C) for a 5-min test session. The duration of immobility was measured upon viewing of the video recordings. Immobility was defined as the lack of active movements except from those required for floating.

### Tissue collection

Mice allocated to immunofluorescence studies were deeply anesthetized with a mixture of xylazine-ketamine (10 mg/g bw, Sanofi) and were perfused transcardially with a solution containing 0.9% NaCl, followed by 4% paraformaldehyde (PFA, 4 °C) in phosphate buffer (pH 7.3). Animals were sacrificed in randomized order to minimize experimental bias. Mice were then decapitated and the brain was carefully removed. Mice allocated to molecular biology analysis, were deeply anesthetized with xylazine-ketamine (10 mg/g bw, Sanofi). The whole HPC was carefully dissected, immediately snap-frozen in liquid-nitrogen, and maintained at −80 °C until further processing. Fecal samples were harvested at the end of the UCMS protocol in donor mice and upon exit from the isolators in recipient mice.

### Quantitative RT-qPCR

Total RNA was extracted from brain tissue and cDNA was synthesized using a Cells-to-Ct Kit (Applied Biosystems) according to the manufacturer’s instructions. Real-time PCR was performed using the SYBR Green Master Mix (Applied Biosystems) and products were detected on an Applied Biosystems ViiA 7 Real-Time PCR System. Relative expression of *Th, Tph2, Ddc, GluR1, Gls1, Gls2, Grid1, Gad1, Gad2, Gabra2* and *Gabrb2* was calculated using the 2(11 C.t/) method. Conditions for real-time PCR were: initial denaturation for 10 min at 95 °C, followed by amplification cycles with 15 s at 95 °C, and 1 min at 60 °C.

### Western blots

Brain tissue was homogenized in RIPA lysis buffer (25 mM Tris-HCl pH 7.6, 150 mM NaCl, 1% NP-40, 1% sodium deoxycholate, 0.1% SDS) (Pierce Thermo Scientific), supplemented with protease (cOmplete, Sigma) and phosphatase (phosSTOP, Sigma) inhibitors. Protein concentration was measured with a Pierce BCA protein Assay Kit (ThermoFischer Scientific) prior to the Western blot assay. Tissue lysates were mixed with 4× NuPage LDS loading buffer (Invitrogen) and reducing agent (Invitrogen NP0004), and proteins were separated on a 12% SDS-polyacrylamide gel (Invitrogen NP0329) and subsequently transferred by semi-dry or liquid transfer onto a PVDF membrane (Trans-blot Turbo Mini PVDF, Biorad). The blots were blocked in 5% BSA in Tris-buffered saline with Tween (TBS-T) and incubated with the primary antibodies, including rabbit polyclonal anti-cFos (Merck, Cat No. ABE457), goat anti-COX2 (abcam, Cat No. ab179800), mouse monoclonal anti-CREB-1 (Santa-Cruz, Cat No. sc-240), rabbit polyclonal anti-phosphoCREB (Merck, Cat No SAB1306301), rabbit polyclonal anti-Cx3cr1 (abcam, Cat No. ab8020), chicken polyclonal anti-DCX (abcam), rabbit polyclonal anti-DCX (abcam, Cat No ab153668), rabbit polyclonal anti-Iba1 (Wako, Cat No 019-19741), mouse polyclonal anti-IL1β/IL-1F2 (R&D Systems, Cat No AF-401-SP), rabbit polyclonal anti-IL6 (abcam, Cat No. ab208113), rabbit polyclonal anti-Ki67 (abcam, Cat No. ab15580), mouse monoclonal anti-Sox2 (abcam, Cat No. ab79351), rabbit polyclonal anti-TGFβ 1 (abcam, Cat No. ab92486), rabbit polyclonal anti-TNFα (abcam, Cat No. ab66579). To detect protein signal, the following Horseradish peroxidase–conjugated secondary antibodies were used: Goat Anti-Rabbit IgG (H + L)-HRP Conjugate (1:6000, Biorad, Cat No. 1706515) and Goat Anti-Mouse IgG1 heavy chain (HRP) (1:6000, abcam, Cat No. ab97240) and rabbit anti-goat IgG (H + L)-HRP (1:6000, Invitrogen, Cat No. 31402). Chemiluminescence detection of proteins was performed with Luminata Crescendo Western HRP Substrate (Merck Millipore) in a Chemidoc Imaging System (Biorad). Bands were quantified using the Image Lab software.

### Immunofluorescence

Perfused brains were cut at 40-micron thick coronal sections using a vibrating microtome (VT1000S, Leica). Immunostaining was performed on free-floating sections. Non-specific staining was blocked by 0.25% Triton and 10% donkey serum albumin (Sigma-Aldrich). Sections were incubated overnight with the following primary antibodies at 4 °C: rabbit anti-DCX (1:400, abcam, Cat No. ab18723), chicken anti-DCX (1:400, abcam, Cat No. ab1536668), rabbit anti-Ki67 (1:200, abcam, Cat No. ab15580), rabbit c-Fos (1/1500, Merck-Millipore, Cat No. ABE457). Sections were incubated with secondary antibodies (Alexa-conjugated secondary antibodies at 1:800 dilution, Jackson ImmunoResearch Laboratories) for 1 H at room temperature. Fluorescent sections were stained with the nuclear dye Hoechst and then mounted using the Fluoromount aqueous mounting medium (Sigma-Aldrich).

### Imaging and quantification

Images were acquired using a confocal laser-scanning microscope (LSM 710, Zeiss) and an Apotome, with Zen Imaging softwares (Zeiss). Z-stacks of the dentate gyrus were obtained (step size: 0.5–1 μm) using sequential scanning. Cell counting was performed either manually or by using the Icy open-source platform (http://www.icy.bioimageanalysis.org). Values are expressed as the mean number of immune-positive cell counts in 8–10 sections per animal.

### Microbial extraction and gut microbiota profiling

Fecal samples were harvested 7 weeks post-inoculation. They were collected in autoclaved Eppendorf tubes and stored at −80 °C until further processing. Cellular DNA was extracted using the QIAamp DNA Stool Mini Kit (Qiagen, Cat# 51504) according to the manufacturer’s instructions and including a step of bead beading to optimize the DNA outcome. The DNA was amplified by means of PCR, using primers specific to the V3 region of the 16 S rRNA gene. Amplicons were randomized and thereafter analyzed by denaturing gradient gel electrophoresis (DGGE) using an acrylamide gel containing a 25–65% chemical gradient (urea and formamide) that enabled separation of the PCR amplicons based on sequence differences in the V3 region. The DGGE profiles were analyzed in Bionumerics Version 4.5 (Applied Maths) using a mix of DNA profiles as a marker.

### 16S sequencing data analysis

Sequences were clustered into operational taxonomic units (OTU) and annotated with the MASQUE pipeline (https://github.com/aghozlane/masque). The final list of 16S rRNA targeted amplicons were converted into the negative logarithm of the expressions of the bacterial taxa (phylum and family). Final data were expressed as the average of the negative logarithm of OTU expression across experimental conditions. All data indexing, segregation and plotting were performed using custom-made scripts in Matlab (Mathworks Inc., 2015 release). Further statistical tests were conducted in the Prism software (GraphPad, version 6, San Diego, USA). The microbiota profiles obtained were further characterized using principal coordinate analysis (PCoA), relative abundance plots and Shannon Index as previously described [3]. Codes are available upon request: https://github.com/SohamSahaNeuroscience/Microbiota-analysis.

### Statistical analysis

All data were expressed as mean ± SEM. Statistical analyses were performed using the Prism software (GraphPad, version 9), with *p* < 0.05 considered statistically significant. Data were analyzed using unpaired two-tailed Mann-Whitney test or the two-way ANOVA repeated measures followed by Bonferroni post-hoc test when appropriate. PCA analysis was performed using the Permanova test. Data met the assumptions of the tests applied and variance was similar across the different groups in different experimental conditions. Subjects were excluded from behavioral assessment when they were not fit or able to finish the trial (for instance risk of drowning in the swim tank or managing to detach the tail from the tail suspension test). Outliers were excluded using the ROUT method (GraphPad Prism, version 9). A detailed statistical analysis is presented in the Supplementary Materials.

## Results

### Inoculation with stress-triggered gut microbiota activates the vagus nerve

**Fig. 1:**
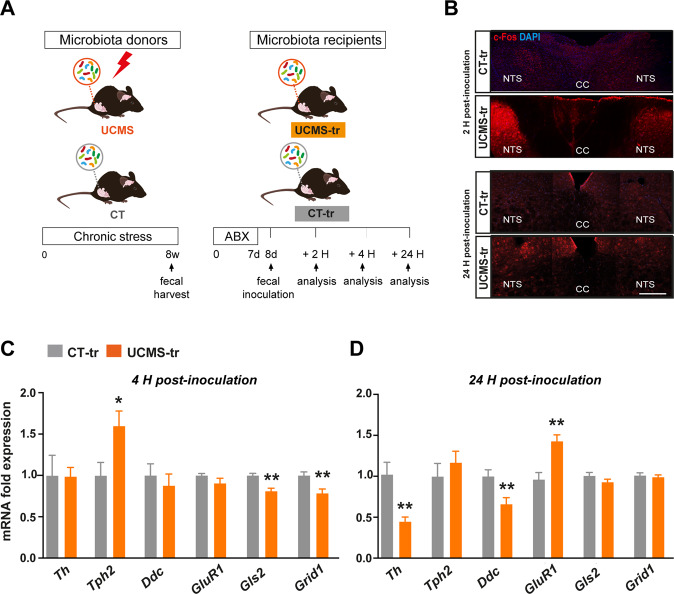
Transfer of gut microbiota from chronically stressed mice induces a rapid activation of the vagus nerve and changes in neurotransmitter pathways in the brainstem. **A** Experimental design and experimental groups. **B** Representative pictures of c-Fos immunostaining in the nucleus of the solitary tract (NTS) in mice inoculated with gut microbiota harvested from either CT (CT-tr group) or UCMS (UCMS-tr group) mice, at 2 H and 24 H post-inoculation. **C**, **D** *Th, Tph2, Ddc, GluR, Gls2 and Grid1* relative expression (RT-qPCR performed in triplicate) in the brainstem of mice at 4 H and 24 H following gut microbiota inoculation. Quantification of mRNA expression is relative to CT-tr mice. Data are shown as mean ± SEM, *n* = 5/group. *p < 0,05 and **p < 0.01. Data were analyzed using the non-parametric Mann-Whitney test. For the 4 H time-point, CT-tr *vs* UCMS-tr: *Th* (*p* = 0.84, U = 11), *Tph2* (*p* = 0.03, U = 2), *Ddc* (*p* = 0.42, U = 8*), GluR1* (*p* = 0.22, U = 6), *Gls2* (*p* = 0.008, U = 0), *Grid1* (*p* = 0.01, U = 1). For the 24 H time-point, CT-tr *vs* UCMS-tr: *Th* (*p* = 0.01, U = 0), *Tph2* (*p* = 0.55, U = 9), *Ddc* (*p* = 0.01, U = 1*), GluR1* (*p* = 0.01, U = 0), *Gls2* (*p* = 0.30, U = 7), *Grid1* (*p* = 0.84, U = 11).

We used mice (8 weeks old, C56BL6/j) that sustained 8 weeks of UCMS and their controls (CT) as GM donor mice (Fig. 1A). At the end of the UCMS protocol, fresh fecal samples were harvested from the donor mice and were transferred by oral gavage to GM recipient mice (8 weeks old, C56BL6/j). To determine whether inoculation with stress-derived GM activates the VN, we looked at c-Fos immunoactivity patterns in the NTS, a standard proxy of VN activation [29]. We found that UCMS-tr mice displayed significant c-Fos immunoreactivity at 2 H, as previously described [29], and this pattern was sustained up to at least 24 H post-inoculation (Fig. 1B). To further validate our results, we performed RT-qPCR analysis using brainstem samples of CT-tr and UCMS-tr mice and found an increase in *cfos* expression in UCMS-tr mice (Supplementary Fig. 1A). We then asked whether inoculation with stress-derived GM would alter the gene expression of key rate limiting enzymes involved in major neurotransmitter pathways in the brainstem, namely serotonin, dopamine, GABA and glutamate. RT-qPCR analysis showed that UCMS-tr mice displayed an increase in brainstem expression of tryptophan hydroxylase (*Tph2*) accompanied by a decrease in glutaminase 2 (*Gls2*) and glutamate ionotropic receptor delta type subunit 1 (*Grid1*) expression at 4 H post-inoculation (Fig. 1C). However, these swift changes were not sustained at 24 H post-inoculation, when we observed a significant decrease in the expression of tyrosine hydroxylase (*Th*) and dopa decarboxylase (*Ddc*), the rate-limiting enzymes involved in the biosynthesis of catecholamines such as dopamine and serotonin. Meanwhile, the specific expression of glutamate receptor 1 (*GluR1*) was significantly increased (Fig. 1D). In contrast, the expression of other glutamate-related and GABA-related enzymes and receptors was not altered (Supplementary Fig. 1B), suggesting that serotonin and dopamine neurotransmission pathways in the brainstem could be preferentially impaired following inoculation with gut microbiota from UCMS-tr mice.

### Stress-triggered gut microbiome activates dentate gyrus neurons and decreases adult hippocampal neurogenesis

**Fig. 2:**
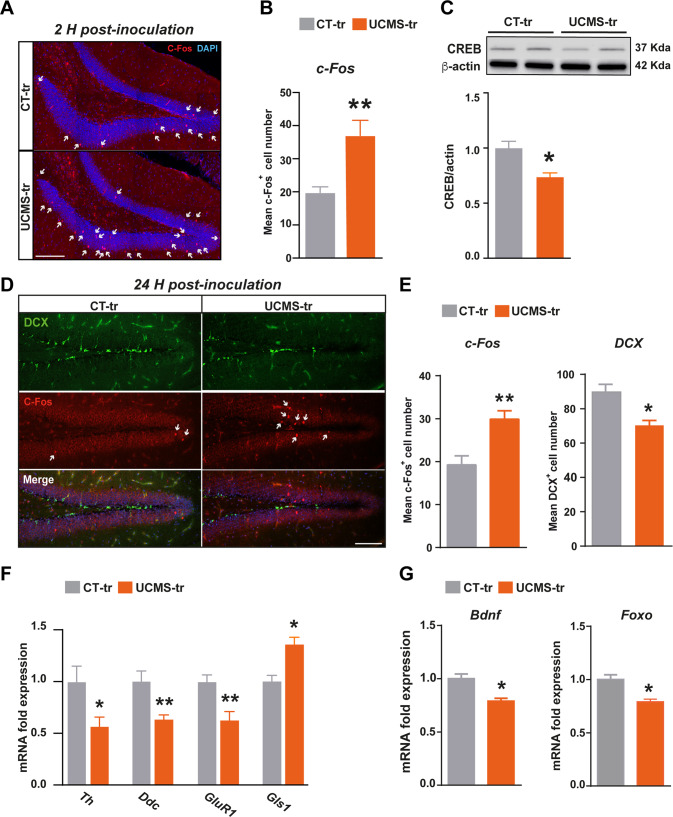
Stress-triggered gut microbiota induce changes in the levels of neurogenic factors and adult hippocampal neurogenesis in recipient mice. **A** Representative pictures of c-Fos immunostaining 2 H following fecal transplantation of either CT (CT-tr group) or UCMS (UCMS-tr) gut microbiota. **B** Bar-plot depicting the mean number of c-Fos positive cells in the dentate gyrus at 2 H post-inoculation with CT and UCMS-derived microbiota (*p* = 0.008, U = 0, *n* = 5/group). **C** Western blot and corresponding quantitative bar-plot of CREB protein levels at 24 H post-inoculation (*p* = 0.02, U = 0, *n* = 5/group). **D**, **E** Representative pictures of C-Fos and DCX immunostaining in the dentate gyrus of CT-tr and UCMS-tr mice, and bar-plots depicting respective positive-cell counting (*p* = 0.008, U = 0 and *p* = 0.03, U = 35 respectively, *n* = 5/group). **F** *Th, Ddc, GluR1* an*d Gls1* relative expression (RT-qPCR performed in triplicate) in the HPC of CT-tr and UCMS-tr mice at 24 H post-inoculation. CT-tr *vs* UCMS-tr: *Th* (*p* = 0.05, U = 2), *Ddc* (*p* = 0.008, U = 0*), GluR1* (*p* = 0.008, U = 0), *Gls1* (*p* = 0.03, U = 1), *n* = 5/group. **G** *Bdnf* and *Foxo* relative expression (RT-qPCR performed in triplicate) in the HPC at 24 H post-inoculation (*p* = 0.05, U = 3 and *p* = 0.02, U = 0 respectively, *n* = 5/group). Data are shown as mean ± SEM, *n* = 5–6/group. *p < 0,05 and **p < 0.01. Quantification of protein and mRNA expression levels is relative to CT-tr mice. Data were analyzed using the non-parametric Mann-Whitney test.

In light of these results, we sought to determine whether GM-induced VN activation affected neuronal activation patterns in the HPC, by measuring c-Fos protein levels in the HPC of CT-tr and UCMS-tr mice. We found that c-Fos immunoreactivity in the dentate gyrus (DG) of UCMS-tr mice was significantly increased as early as 2 H and at least up to 24 H post-inoculation (Fig. 2A, B, D–E). We corroborated this swift and sustained increase in neuronal activity by performing Western blot analysis on hippocampal lysates (Supplementary Fig. 1C). Interestingly, when we looked at c-Fos expression across the neurogenic DG, we observed that the increase of c-Fos immunoreactivity in UCMS-tr mice was associated with a decrease in the number of doublecortin-positive (DCX^+^) neurons (Fig. E). The pool of DCX^+^ cells in the DG, besides being a step in the maturation process of newborn neurons, is of importance in buffering the negative feedback on the hypothalamic–pituitary-adrenal stress-axis [30]. However, we did not observe any co-localization of c-Fos and DCX, meaning that this increased neuronal activation mainly concerns preexisting neurons and not newly-generated ones. To further investigate the effects of GM inoculation in early HPC responses, we assessed the expression of key enzymes and receptors involved in major neurotransmitter pathways, namely serotonin, dopamine, GABA and glutamate neurotransmission. RT-qPCR analysis showed that UCMS-tr mice displayed a significant decrease in *Th* and *Ddc* expression in the HPC, mirroring the early changes observed in the brainstem and indicative of perturbations in serotonin availability and dopamine biosynthesis (Fig. 2F). Moreover, a significant decrease in *GluR1* and *Gad2*, as well as an increase in *Gls1* were observed (Fig. 2F, Supplementary Fig. 1D). The expression of other glutamate-related and GABA-related enzymes and receptors was not affected (Supplementary Fig. 1D).

In view of these results, we measured the levels within the HPC of different factors known to regulate adult HPC neurogenesis and to have an important function in the pathophysiology of depression, namely brain derived neurotrophic factor (BDNF), transcription factor cAMP response element-binding protein (CREB) and Forkhead Box O transcription factor (FOXO). We observed that inoculation with UCMS microbiota significantly decreased all these factors, suggesting a likely implication of these changes in the ensuing adult HPC neurogenesis deficits (Fig. 2C, G).

Taken together, these results indicate that inoculation with a perturbed GM results in increased neuronal activation within the NTS and HPC, associated with a decrease in the expression of key enzymes involved in the biosynthesis of serotonin and dopamine, a decrease in neurogenic factors in the HPC and ultimately a rapid impact on neuronal differentiation within the HPC neurogenic niche. Collectively, these effects could impact some HPC-dependent behaviors.

### Depression-like responses in mice receiving stressed-related microbiome

**Fig. 3:**
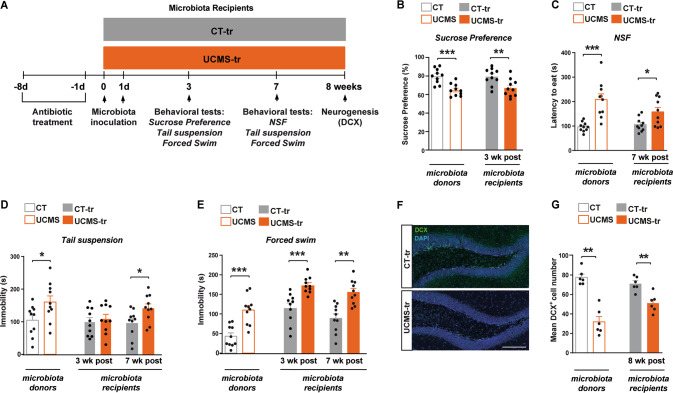
Gut microbiota from chronically stressed mice promotes depressive-like behavior and decreases adult hippocampal neurogenesis in host mice. **A** Experimental timeline. **B** Bar-plots showing the percentage of sucrose preference in microbiota donor and recipient mice (CT *vs* UCMS: *p* = 0.0005, U = 7.5; CT-tr *vs* UCMS-tr: *p* = 0.01, U = 16). **C** Bar-plots depicting the latency to eat in the novelty suppressed feeding test (NSF; CT *vs* UCMS: *p* < 0.0001, U = 2.5; CT-tr *vs* UCMS-tr: *p* = 0.05, U = 24). **D**, **E** Total immobility in the tail suspension and forced swim tests, both indicative of despair-like behaviors. Different cohorts of recipient mice were employed for the assessments at 3 weeks and 7 weeks post-microbiota inoculation [CT *vs* UCMS: Tail suspension test (*p* = 0.02, U = 20.5) and Forced swim test (*p* = 0.0007, U = 8). CT-tr *vs* UCMS-tr: Tail suspension test at 3 weeks post-inoculation (*p* = 0.48, U = 40) and 7 weeks post-inoculation (*p* = 0.03, U = 21). Forced swim test at 3 weeks post-inoculation (*p* = 0.0001, U = 4.5) and 7 weeks post-inoculation (*p* = 0.002, U = 10.5)]. **F**, **G** Representative pictures of DCX^+^ immunostaining in the dentate gyrus of CT-tr and UCMS-tr mice and bar-plot showing the mean number of DCX-positive cells in different experimental groups (CT *vs* UCMS: *p* = 0.002, U = 0; CT-tr *vs* UCMS-tr: *p* = 0.004, U = 1). Data are shown as mean ± SEM. *p < 0.05, **p < 0.01 and ***p < 0.001. Data were analyzed using the non-parametric Mann-Whitney test. NSF novelty suppressed feeding.

We sought to determine whether stress-induced GM imbalance is sufficient to modulate affective behavior, as previously shown [3, 4]. To address this question, we assessed behavior at early and belated time-points following GM inoculation (Fig. 3A). Our results showed that UCMS-tr mice adopted the behavioral phenotype of donor-stressed mice, characterized by depression-like responses. In the sucrose preference test, both UCMS and UCMS-tr mice displayed a significant decrease in sucrose consumption compared to their control counterparts, a standard indication of depressive-like states (Fig. 3B). In the novelty suppressed feeding (NSF) test, both UCMS and the UCMS-tr mice showed a significantly increased latency to eat, reflective of increased anxiety- and depression-like behavior (Fig. 3C). Moreover, in both the tail suspension and forced swim tests, UCMS and UCMS-tr mice displayed more immobility compared to their controls, indicative of depression-like behavior (Fig. 3D–E). To further investigate whether these depressive states correlated with lasting changes in adult HPC neurogenesis, we performed immunostaining for DCX and found that the transfer of stress-derived GM significantly decreased the number of DCX^+^ neurons in the DG, reflective of deficits in the maturation step of adult-born neurons (Fig. 3F–G).

**Fig. 4:**
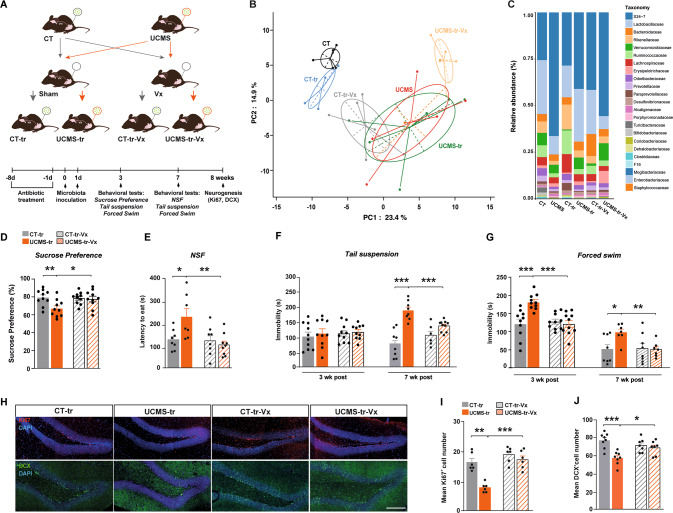
The effects of chronic stress-induced gut microbiota perturbations on emotional behavior and adult hippocampal neurogenesis are mediated by the vagus nerve. **A** Schematic representation of the fecal transplantation paradigm and experimental groups. Mice sustained vagotomy (Vx) 2 weeks prior inoculation with gut microbiota from either control (CT) or UCMS mice. **B** PCoA of 16S rRNA expression data (Canberra distance) showing group differences in bacterial family levels among the different experimental groups (PC1: 23,4% and PC2: 14.9%, Permanova, *p* < 0,001). **C** Bar-plot showing 16 S rRNA expression patterns in a family level. Bacterial families are shown on the right. **D** Bar-plots depicting sucrose preference (CT-tr *vs* UCMS-tr: *p* = 0.01, U = 16.5; UCMS-tr *vs* UCMS-tr-Vx: *p* = 0.04, U = 22.5). **E**–**G** Bar-plots showing the latency to eat the pellet in the novelty suppressed feeding test (CT-tr *vs* UCMS-tr: *p* = 0.03, U = 9.5; UCMS-tr *vs* UCMS-tr-Vx: *p* = 0.005, U = 6) and total immobility in the tail suspension (CT-tr *vs* UCMS-tr at 7 weeks post-inoculation: *p* = 0.0002, U = 0; UCMS-tr -Vx *vs* UCMS-tr-Vx at 7 weeks post-inoculation: *p* = 0.001, U = 6) and forced swim tests (CT-tr *vs* UCMS-tr at 3 weeks post-inoculation: *p* = 0.0001, U = 4.5 and 7 weeks post-inoculation: *p* = 0.03, U = 10. UCMS-tr *vs* UCMS-tr-Vx at 3 weeks post-inoculation: *p* < 0.0001, U = 2 and 7 weeks post-inoculation: *p* = 0.003, U = 4). **H**–**J** Re*p*resentative images and corresponding bar-plots of Ki67^+^ and DCX^+^ cells in the dentate gyrus of CT-tr, UCMS-tr, CT-tr-Vx and UCMS-tr-Vx mice. Scale bars: 100 μm. For CT-tr *vs* UCMS-tr: DCX-positive neurons (*p* = 0.001, U = 3) and Ki-67 positive cells (*p* = 0.004, U = 1). All data are represented as mean ± SEM. *p < 0.05, **p < 0.01, ***p < 0.001. Statistical significance was calculated using the non-parametric Mann-Whitney test. PCA analysis was run by Permanova. NSF novelty suppressed feeding.

Finally, we validated the impact of UCMS on GM composition by performing 16S rRNA gene V3-V4 region sequencing on the fecal samples of GM donor and recipient mice (Fig. 4A–C, Supplementary Fig. 2). Principal-coordinate analysis (PCoA) showed that the CT and UCMS groups clustered differently, as did their recipient counterparts (Fig. 4B, Supplementary Fig. 2A), confirming the impact of UCMS on the gut microbiome as well as the efficacy of the microbial inoculation. Taxonomic analysis further revealed several UCMS-related changes in bacterial families and phyla (Fig. 4C, Supplementary Fig. 2B) and changes in bacterial diversity, depicted by the Shannon Index (Supplementary Fig. 2C).

### The vagus nerve mediates the effects of a stress-triggered gut microbiome on affective behaviors and adult hippocampal neurogenesis

To assess whether the VN transduces the effects of stress-induced GM changes on affective behavior and adult HPC neurogenesis, we employed additional cohorts of animals that had sustained subdiaphragmatic vagotomy (Vx) or sham surgery 2 weeks prior fecal transplantation (Fig. 4A). We found that ablation of the VN abrogated the GM-induced transmission of depressive-like states. More precisely, unlike sham-operated mice, vagotomized mice did not display a decrease in sucrose preference when inoculated with UCMS-microbiota (Fig. 4D). Moreover, inoculation of sham-operated animals with UCMS microbiota significantly increased the latency to eat in the NSF and immobility in the tail suspension and forced swim tests, while these depressive-like responses were absent in vagotomized animals (Fig. 4E–G). Furthermore, Vx protected against the decrease in cell proliferation and neuronal differentiation induced by inoculation with UCMS-harvested GM, as depicted by the numbers of Ki67^+^ and DCX^+^ cells in the DG (Fig. 4H–J). These results indicate that VN integrity is necessary for the transmission of the depression-like phenotype and adult HPC neurogenesis deficits after inoculation with disturbed GM. It is important to note that we did not witness any modifications in anxiety- and depression-like behaviors or body weight by Vx or antibiotic treatment alone (Supplementary Figs. 3, 4, 5A–I).

### Stress-derived gut microbiome induces early and sustained neuroinflammation in a vagus nerve-dependent manner

**Fig. 5:**
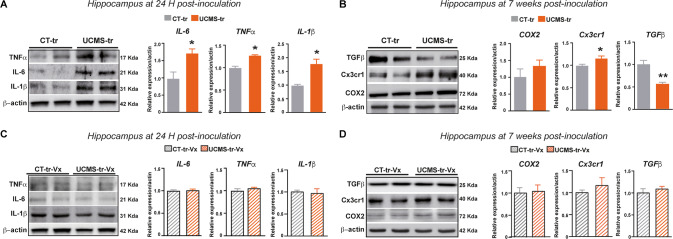
The vagus nerve mediates neuroinflammatory responses in the hippocampus induced by inoculation with a stress-triggered gut microbiome. **A** Western blot membranes and corresponding bar-plots depicting the increase in the protein levels of different neuroinflammatory mediators detected in the hippocampus of UCMS-tr mice, at 24 H post-inoculation. **B** Representative western blots and corresponding bar-plots showing an increase in the protein levels of neuroinflammatory mediators in the hippocampus of UCMS-tr mice at 7 weeks post-inoculation. **C** Western blot membranes and corresponding quantifications of neuroinflammatory mediators in the hippocampus of vagotomized recipient mice at 24 H post-inoculation, showing no differences between the groups. **D** Western blot membranes and corresponding quantifications showing the levels of different neuroinflammatory mediators in the hippocampus of vagotomized recipient mice at 7 weeks post-inoculation. Quantification of protein expression is relative to CT-tr mice. Data are shown as mean ± SEM (*n* = 5/group) and were analyzed using the non-parametric Mann-Whitney test.

Mounting evidence points to a key role of neuroinflammation as a factor that negatively affects adult HPC neurogenesis and that is associated with the pathophysiology of depression [31]. Moreover, emerging evidence points to a key role of neuroinflammation in the inter-communication between GM and the brain via VN afferents [32, 33]. In this framework, we first investigated whether GM-induced VN activation affects neuroinflammation in the HPC. We found that UCMS-tr mice indeed displayed signs of neuroinflammation in the HPC at 24 H post-inoculation, namely an increase in the levels of the pro-inflammatory cytokine tumor necrosis factor alpha (TNFα), interleukin-1 beta (IL-1β) and interleukin 6 (IL-6) (Fig. 5A). This neuroinflammatory state was sustained up to at least 7 weeks post-inoculation, when we found a significant increase in microglial cell mobilization marker Cx3cr1 and a slight yet non-significant increase in COX2, accompanied by a decrease in pro-neurogenic transforming growth factor beta (TGFβ) (Fig. 5B). Importantly, when we examined the levels of pro-inflammatory mediators and pro-neurogenic factors in inoculated mice that had sustained Vx, we found no difference between the groups (Fig. 5C, D, Supplementary Fig. 5J), suggesting that VN integrity is required to mediate the effects of a disturbed microbiome on neuroinflammation.

## Discussion

The VN acts as a conduit ferrying signals from the gut to the brain and vice versa, however the extent of its implication in the communication between the GM and the brain and the mechanisms underpinning this interplay are still under active investigation. The present study demonstrates that chronic stress-induced GM perturbations activate the VN and induce prompt serotonin and dopamine neurotransmission deficits in the brainstem and HPC, early and belated neuroinflammation and impairments in adult HPC neurogenesis that are ultimately associated with well-seated depressive states in recipient mice. We found that GM changes require an intact VN to produce these effects, suggesting that vagal afferents are major components of the GM-brain regulation system and potential targets of therapeutic intervention for stress-related disorders, including depression.

Our results support previous studies showing altered cecal and fecal microbiota composition in experimental models of stress and depressed subjects [3, 4, 34–36]. These GM alterations may contribute to the neuro-progression of stress-related depression by altering physiological processes that depend on vagal afferents, such as neurotransmitter release and immunity/inflammation [37–39]. Vagal afferent terminals are located within the gastrointestinal lamina propria where they can directly or indirectly interact with the microbiome and sense immune or endocrine signals originating from the GI tract [40]. Our results show that inoculation with GM from chronically stressed mice induces a rapid activation of the VN, accompanied by a decrease in rate-limiting enzymes implicated in the biosynthesis and availability of dopamine and serotonin, suggesting perturbations in serotonin and dopamine neurotransmission. Indeed, VN activation has been linked to serotonin release, since the VN sends direct projections to the LC and DRN, which are hubs for serotonergic neurons [41]. Serotonergic neurons in the DRN project axons into several forebrain areas including the HPC, meaning that changes in serotonin bioavailability within the brainstem could directly impact the HPC. Through virus-based tracing techniques, it has been shown that vagal signals derived from the gut are relayed from the NTS through the medial septum to hippocampal glutamatergic neurons, allowing gut-derived signals transmitted through the VN to control HPC-dependent functions and reduce the expression of neurogenic and neurotrophic markers [10].

In our experimental paradigm, we show that the HPC is quickly affected following inoculation with a disturbed gut microbiome, demonstrated by an increase in c-Fos expression and a decrease in DCX protein levels in the neurogenic DG, reflective of deficits in the maturation process of adult-born neurons. Importantly, adult neurogenesis in the DG is a well-documented cellular substrate for both the pathophysiology and treatment of depressive disorders [12–14]. However, it is important to note here that although DCX has been extensively used as a standard neurogenesis marker [42] and for migrating neuronal progenitor cells [43], other studies argue that DCX expression alone could be disconnected from neurogenesis. This position is corroborated by reports showing that DCX KO mice display normal levels of neurogenesis [44], and that maturation of adult-born neurons is partially independent of the proliferation and survival of these cells [45]. Nevertheless, using our experimental paradigm, we have previously shown that inoculation with UCMS-derived GM not only reduces the number of proliferating cells, but it also decreases the number of EdU-positive “surviving” cells in the DG (with EdU being injected 3 weeks before sacrifice) [4], suggesting that our model induces a deficit in all the well-documented steps of adult neurogenesis, namely proliferation, differentiation and maturation of adult-born neurons. Moreover, it is to be noted that the inoculation-induced effects on adult neurogenesis could also affect another well-documented neurogenic niche of the brain, the subventricular zone, and may impact associated functions, such as olfactory perception, acuity and memory. This possibility is not addressed herein and could constitute the scope of a future study.

The early consequences of GM inoculation on the HPC could be mediated, at least in part, by changes in serotonin and dopamine bioavailability and on the levels of neurogenic factors within the HPC. Indeed, it is widely accepted that changes in synaptic serotonin, dopamine and BDNF levels are coupled with altered synaptic plasticity and HPC neurogenesis [46]. Our analysis confirmed that, similarly to what observed in the brainstem, *Th* and *Ddc* gene expression levels are also decreased in the HPC, as are some key transcriptional factors linked to adult neurogenesis, namely CREB, *Foxo* and *Bdnf*. Although a wide spectrum of processes is controlled by these factors, their implication in the regulation of synaptic plasticity is the most extensively reported. Moreover, antidepressants indirectly regulate various factors involved in cell survival pathways, including CREB and BDNF [47].

Our results also show that inoculation with UCMS-derived GM leads to early and sustained neuroinflammatory responses in the HPC. In fact, numerous studies have described the existence of a strong association between depression and peripheral markers of inflammation in both blood and cerebrospinal fluid [48, 49]. Moreover, both clinical and preclinical evidence suggests that neuroinflammation is a key factor that interacts with most neurobiological correlates of major depressive disorder, namely depletion of brain serotonin, dysregulation of the HPA axis and alteration of adult HPC neurogenesis [31]. Interestingly, it has been reported that the VN can regulate neuroinflammatory responses in the CNS. For instance, it has been reported that acute or chronic VN stimulation increases the expression of IL‐1β in the hypothalamus and HPC [50, 51]. Our results strongly suggest that VN integrity is required for the transmission of the GM-driven imprint on cellular homeostasis and behavior. Importantly, clinical studies have shown that VN stimulation (VNS) exerts positive results in treatment resistant depression, with response and remission rates increasing further with longer VNS treatments [52–54]. These results are corroborated by animal models, where it has been demonstrated that manipulation of VN activity significantly attenuates depressive symptoms and HPC neurogenesis deficits [55–57]. Importantly, VNS therapy is now approved for treatment-resistant depression in the European Union, the United States and Canada, although its precise mechanisms of action are still elusive. It is worth noting that there is a growing body of observational data in humans, from Danish and Swedish healthcare registries, that demonstrates an association between vagotomy and a reduced risk for the development of Parkinson’s disease [58], highlighting the importance of the VN circuitry in the pathophysiology and treatment of not only depression, but also neurodegenerative disorders.

Our results are in line with previous findings showing that the VN mediates the effects of some probiotic strains on stress responses in rodents [7–9] and on neurotransmission and neuroplasticity [11, 57, 59, 60]. However, our study does not allow to pinpoint specific bacterial strains that could impact the brain via a VN-HPC circuit. Future studies could harness next-generation functional CNS circuit mapping methods, such as viral optogenetic tracing coupled with electrophysiology, to identify the specific neural circuits arising from the brainstem to the HPC that are activated following UCMS-microbiota inoculation. Moreover, coupling DNA sequencing to metatranscriptomics, metabolomics, lipidomics and proteomics could help unravel molecular mediators of the GM–VN-brain axis. Another weakness of our study is the use of subdiaphragmatic vagotomy as a means to abolish VN activity. Virus-based methods should be preferentially used instead to optogenetically or chemogenetically inhibit or activate VN afferents selectively and study the impact on behavior and adult neurogenesis [51]. Finally, it is worth highlighting that not all microbial signals to the brain are mediated by the VN. For instance, anxiety-like behavior in mice induced by a mild gastrointestinal infection is still evident after vagotomy [61], which indicates that other biological pathways (some of which are known to be influenced by the microbiota) can mediate the anxiogenic effects of gut microbiota, such as microbial metabolites or byproducts and immune mechanisms.

Manipulating the GM-VN-brain pathway by either activating the VN or modifying the gut microbiota has become an exciting opportunity in the quest for developing alternative therapies for treatment resistant depression. Dosing trials of VNS therapy in treatment-resistant depression are ongoing [62], and the findings of these studies in conjunction with the cumulative experience by clinicians will determine future therapeutic choices. In addition to representing a novel therapeutic modality, VNS therapy is a research tool that offers the hope of better understanding the mechanisms underlying depression. Here, we present further arguments in support of the emerging hypothesis that GM can influence brain function and affect emotional states directly through the secondary afferent projections of the VN to the HPC.

### Supplementary information

Supplemental Information

Supplemental Figure 1

Supplemental Figure 2

Supplemental Figure 3

Supplemental Figure 4

Supplemental Figure 5

Supplemental Table
