# Supplementary material for: Gut microbiota changes require vagus nerve integrity to promote depressive-like behaviors in mice
Source: Mol Psychiatry. 2023 May 2;28(7):3002–12. doi: 10.1038/s41380-023-02071-6 (PMC10615761; doi:10.1038/s41380-023-02071-6)
Supplement: Supplementary file 1 — Supplemental Information [file 41380_2023_2071_MOESM1_ESM.pdf]

SUPPLEMENTARY MATERIALS

Quantitative RT-qPCR

Total RNA was extracted from brain tissue and cDNA was synthesized using a Cells-to-Ct Kit (Applied Biosystems) according to the manufacturer's instructions. Real-time PCR was performed using SYBR Green Master Mix (Applied Biosystems) and products were detected on an Applied Biosystems ViiA 7 Real-Time PCR System. Relative expression of *Bdnf, Creb, Cfos*, *Ddc, Foxo, Gad1, Gad2, Gabra2, Gabrb2*, *GluR1, Gls1, Gls2, Grid1, Th* and *Tph2* was calculated using the 2(11C.t/) method. Data were normalized with the house-keeping gene and with controls. Conditions for real-time PCR were: initial denaturation for 10 min at 95°C, followed by amplification cycles with 15 s at 95°C, and 1 min at 60°C. RT-PCR primers used were: Bdnf primer Forward 5'-TCATACT TCGGTTGCATGAAGG-3', Bdnf primer Reverse 5'-AGACCTC TCGAACCTGCCC-3', cFos primer Forward 5'-CAGAGCG CAGAGCATCGCCA-3', cFos primer Reverse 5'-CGATTCC GCCACTTGGCTGC-3', Creb primer Forward 5'-AGCAGCT CATGCAACATCATC-3', Creb primer Reverse 5'-AGTCCTT ACAGGAAGACTGAACT-3', Ddc primer Forward 5'-AGCTGGT TGCTTACACATCTG-3', Ddc primer Reverse 5'-CGAAGGG ACTGCTTTTAGCTT-3', Foxo primer Forward 5'-ATGCTCA ATCCAGAGGGAGG-3', Foxo primer Reverse 5'-ACTCGCA GGCCACTTAGAAAA-3', Gabra2 primer Forward 5'-GGAA GCTACGCTTACACAACC-3', Gabra2 primer Reverse 5'-CCATC GGGAGCAACCTGAA-3', Gabrb2 primer Forward 5'-AAAG GGGCTACTTTGGGATTTG-3', Gabrb2 primer Reverse 5'-ACCAGCGACATATTACTAGGGTC-3', Gad1 primer Forward 5'-CCACCA, AGGTTCTGGATTTCC-3', Gad1 primer Reverse 5'-GTACTTC

AGGGTGTCTCTACAGT-3’, Gad2 primer Forward 5’-TCCGGC TTTTGGTCCTTCG-3’, Gad2 primer Reverse 5’-ATGCCGC CCGTGAACTTTT-3’, Gls1 primer Forward 5’-GACAACGTCAGATGGTGTCAT-3’, Gls1 primer Reverse 5’-TGCTTGT GTCAACAAAACAATGT-3’, Gls2 primer Forward 5’-CTACAGG ATTGCGAACATCTGAT-3’, Gls2 primer Reverse 5’-ACACCAT CTGACGTTGTCTGA-3’, Glur1 primer Forward 5’-AAAGGA GTGTACGCCATCTTTG-3’, Glur1 primer Reverse 5’-TGTCAAC GGGAAAACTTGGAG-3’, Grid1 primer Forward 5’-GGATAT GCCAGTGCGTTACG-3’, Grid1 primer Reverse 5’-GAACAC CCTGTCGTCCTTGG-3’, Th primer Forward 5’-GTACGGGT CAAACTTCACAG-3’, Th primer Reverse 5’-CAGCCCTAC CAAGATCAAC-3’, Tph2 primer Forward 5’-GTGACC CTGAATCCGCCTG-3’, Tph2 primer Reverse 5’-GGTGCCG TACATGAGGACT-3’.

Statistical analysis

A detailed presentation of the statistical analysis performed is presented below.

mRNA expression of key neurotransmission players in the brainstem at 4 H and 24 H post-inoculation (Figure 1C-D and Supplementary Figure 1B)

Analysis was performed using the Mann-Whitney test (n=5/group). For the 4 H time-point, CT-tr *vs* UCMS-tr: *Th* (*p*=0.84, U=11), *Tph2* (*p*=0.03, U=2), *Ddc* (*p*=0.42, U=8), *GluR1* (*p*=0.22, U=6), *Gls2* (*p*=0.008, U=0), *Grid1* (*p*=0.01, U=1). For the 24 H time-point, CT-tr *vs* UCMS-tr: *Th* (*p*=0.01, U=0), *Tph2* (*p*=0.55, U=9), *Ddc* (*p*=0.01, U=1), *GluR1* (*p*=0.01, U=0), *Gls2* (*p*=0.30, U=7), *Grid1* (*p*=0.84, U=11), *Gad1* (*p*=0.84, U=11), *Gad2* (*p*=0.42, U=8), *Gls1* (*p*=0.22, U=6), *Gabra2* (*p*=0.84, U=11), *Gabrb2* (*p*=0.55, U=9).

CT-tr vs UCMS-tr mRNA expression of key neurotransmission players in the hippocampus at 24 H post-inoculation (Figure 2F and Supplementary Figure 1D)

Analysis was performed using the Mann-Whitney test (n=5/group), CT-tr *vs* UCMS-tr: *Th* (*p*=0.05, U=2), *Ddc* (*p*=0.008, U=0*), GluR1* (*p*=0.008, U=0), *Gls1* (*p*=0.03, U=1), *Gad1* (*p*=0.56, U=7), *Gad2* (*p*=0.05, U=4), *Gls2* (*p*=0.90, U=9), *Gabra2* (*p*=0.73, U=8), *Gabrb2* (*p*=0.55, U=7).

Protein and mRNA expression of c-FOS and neurogenic factors in the brainstem and hippocampus (Figure 2C,G and Supplementary Figure 1A,C)

Analysis was performed using the Mann-Whitney test (n=5/group). For the brainstem, CT-tr *vs* UCMS-tr: *cfos* mRNA expression at 4 H and 24 H: *p*=0.02, U=0 and *p*=0.02, U=1 respectively. For the hippocampus, CT-tr *vs* UCMS-tr: c-Fos protein expression at 4 H and 24 H (*p*=0.03, U=0 and *p*=0.03, U=0), CREB (*p*=0.02, U=0), *Bdnf* (*p*=0.05, U=3), *Foxo* (*p*=0.02, U=0), c-Fos positive cells at 2 H post-inoculation (*p*=0.008, U=0) and 24 H post-inoculation (*p*=0.002, U=20), DCX-positive neurons at 24 H post-inoculation (*p*=0.03, U=35).

Behavioral analysis at 3 and 7 weeks post-inoculation (Figures 3B-E and 4D-G)

Analysis was performed using the Mann-Whitney test (n=10/group). Below are the group comparisons.

- CT *vs* UCMS:

Sucrose preference test (*p*=0.0005, U=7.5)

Tail suspension test (*p*=0.02, U=20.5)

Forced swim test ($p=0.0007$, U=8)

Novelty suppressed feeding ($p<0.0001$, U=2.5).

- CT-tr *vs* UCMS-tr

Sucrose preference test ($p=0.01$, U=16.5)

Novelty suppressed feeding ($p=0.05$, U=24 in Figure 3C and $p=0.03$, U=9.5 in Figure 4G)

Tail suspension test at 3 weeks post-inoculation ($p=0.48$, U=40) and 7 weeks post-inoculation ($p=0.03$, U=21 in Figure 3D and $p=0.0002$, U=0 in Figure 4F)

Forced swim test at 3 weeks post-inoculation ($p=0.0001$, U=4.5) and 7 weeks post-inoculation ($p=0.002$, U=10.5 in Figure 3E and $p=0.03$, U=10 in Figure 4G).

- CT-tr-Vx *vs* UCMS-tr-Vx

Sucrose preference test ($p=0.89$, U=48)

Novelty suppressed feeding ($p=0.62$, U=30.5)

Tail suspension test at 3 weeks post-inoculation ($p=0.75$, U=45.5) and 7 weeks post-inoculation ($p=0.06$, U=13)

Forced swim test at 3 weeks post-inoculation ($p=0.64$, U=43.5) and 7 weeks post-inoculation ($p=0.87$, U=30).

UCMS-tr *vs* UCMS-tr-Vx

Sucrose preference test ($p=0.04$, U=22.5)

Novelty suppressed feeding ($p=0.005$, U=6)

Tail suspension test at 3 weeks post-inoculation ($p=0.86$, U=47.5) and 7 weeks post-inoculation ($p=0.001$, U=6)

Forced swim test at 3 weeks post-inoculation (*p*<0.0001, U=2) and 7 weeks post-inoculation (*p*=0.0033, U=4).

Neurogenesis analysis at 8 weeks post-inoculation (Figures 3F-G and 4H-J)

Analysis was performed using the Mann-Whitney test (n=6/group). Below are the group comparisons.

- CT *vs* UCMS : DCX-positive neurons (*p*=0.002, U=0)
- CT-tr *vs* UCMS-tr : DCX-positive neurons (*p*=0.004, U=1) ; Ki-67 positive cells (*p*=0.004, U=1)
- CT-tr-Vx *vs* UCMS-tr-Vx : DCX-positive neurons (*p*=0.75, U=13) ; Ki-67 positive cells (*p*=0.24, U=8).

16s-RNA sequencing analysis (Figure 4B-C and Supplementary Figure 2)

PCA analysis was performed with Permanova (*p* <0.001 and *p*<0.0001 for genus and family levels respectively), n=5-6/group. Shannon Index analysis was performed with Mann-Whitney, as follows: Family: CT *vs* UCMS (*p*=0.0003), CT-tr *vs* UCMS-tr (*p*=0.03), CT-tr-Vx *vs* UCMS-tr-Vx (*p*=0.31); genus: CT *vs* UCMS (*p*=0.96), CT-tr *vs* UCMS-tr (*p*=0.97), CT-tr-Vx *vs* UCMS-tr-Vx (*p*=0.73); phylum: CT *vs* UCMS (*p*=0.81), CT-tr *v*s UCMS-tr (*p*=0.20), CT-tr-Vx *vs* UCMS-tr-Vx (*p*=0.98).

Analysis of neuroinflammatory mediators in the hippocampus (Figure 5)

Data were analyzed by the Mann-Whitney test (n=5/group), as follows:

- CT-tr *vs* UCMS-tr: IL-6 (*p*=0.05, U=1), TNFα (*p*=0.03, U=0), IL-1β (*p*=0.02, U=0), COX2 (*p*=0.19, U=4), Cx3cr1 (*p*=0.05, U=13), TGFβ (*p*=0.01, U=0).

- CT-tr-Vx *vs* UCMS-tr-Vx: IL-6 (p=0.90, U=9), TNFα (p=0.41, U=6), IL-1β (p=0.73, U=8), COX2 (p=0.90, U=9), Cx3cr1 (p=0.41, U=6), TGFβ (p=0.41, U=6), BDNF (p=0.28, U=5), pCREB/CREB (p=0.28, U=5).

Longitudinal body weight analysis (Supplementary Figure 3)

The two-way ANOVA (repeated measures) was used (n=20/group):

- CT *vs* UCMS (F = 0.69, p=0.5585)
- CT-tr, UCMS-tr, CT-tr-Vx and UCMS-tr-Vx (F= 0.32, p=0.9927)

Effect of antibiotics (ABX) and vagotomy (Vx) on emotional behavior (Supplementary Figures 4 and 5)

- Tail suspension test (ABX *vs* vehicle: p=0.34, U=37; Vx *vs* Sham: p=0.59, U=34)
- Forced swim test (ABX *vs* vehicle: p=0.64, U=43.5; Vx *vs* Sham: p=0.35, U=19.5)
- Elevated Plus maze (ABX *vs* vehicle: p=0.94, U=39.5; Vx *vs* Sham: p=0.86, U=38).

FIGURE FOR SUPPLEMENTARY TABLE 1

Supplementary Table 1.

Table depicting an overview of the experimental sets used in this study, complete with the allocated groups, number of subjects attributed to each group and type of analysis performed.

LEGENDS FOR SUPPLEMENTARY FIGURES

Supplementary Figure 1

(A) *Cfos* relative expression (RT-qPCR performed in triplicate) in the brainstem at 4 H and 24 H following inoculation with either CT- or UCMS-derived gut microbiota. **(B)** Relative expression (RT-qPCR performed in triplicate) of enzymes and receptors implicated in key neurotransmitter pathways in the brainstem of inoculated mice. **(C)** Western blot and corresponding bar-plot of C-Fos protein levels in the hippocampus at 4 H and 24 H post-inoculation with either CT or UCMS microbiota. Quantification of protein and mRNA expression is relative to CT-tr mice. **(D)** Relative expression (RT-qPCR performed in triplicate) of enzymes and receptors implicated in key neurotransmitter pathways in the hippocampus of mice inoculated with either CT- or UCMS-derived gut microbiota. Data are shown as mean ± SEM, n=5/group. Data were analyzed using the non-parametric Mann-Whitney test. For the brainstem, CT-tr *vs* UCMS-tr: *Gad1* ($p=0.84$, U=11), *Gad2* ($p=0.42$, U=8), *Gls1* ($p=0.22$, U=6), *Gabra2* ($p=0.84$, U=11), *Gabrb2* ($p=0.55$, U=9). For the hippocampus, CT-tr *vs* UCMS-tr: *Th* ($p=0.05$, U=2), *Ddc* ($p=0.008$, U=0), *GluR1* ($p=0.008$, U=0), *Gls1* ($p=0.03$, U=1), *Gad1* ($p=0.56$, U=7), *Gad2* ($p=0.05$, U=4), *Gls2* ($p=0.90$, U=9), *Gabra2* ($p=0.73$, U=8), *Gabrb2* ($p=0.55$, U=7).

Supplementary Figure 2

(A) PCoA of 16S rRNA expression data (Canberra distance) showing group differences in bacterial genus levels among the different experimental groups (PC1: 20,9% and PC2: 13.9%; $P<0,001$). **(B)** Bar-plot showing 16S rRNA expression patterns in a phylum level. Bacterial families are shown in the right. **(C)** Bar plots showing the

Shannon Index across experimental groups in a family, genus and phylum level. Data are shown as mean ± SEM, n=5/group. Data were analyzed using the non-parametric Mann-Whitney test.

Supplementary Figure 3

Longitudinal body weight measurements of gut microbiota donor (CT and UCMS) and recipient mice that either sustained (CT-tr+Vx, UCMS-tr+Vx) or did not sustain (CT-tr, UCMS-tr) supradiaphragmatic vagotomy (Vx). Data were analyzed using the non-parametric Mann-Whitney test or two-way ANOVA test followed by Bonferroni post-hoc when appropriate. N=10/group.

Supplementary Figure 4.

(A) Effects of a 7-day antibiotic treatment (ABX) on body weight in sham-operated and vagotomized (Vx) mice. **(B-C)** Effects of a 7-day ABX cocktail on cecum weight and representative cecum pictures. **(D-F)** Impact of a 7-day ABX treatment on standard readouts for anxiety-like (Elevated plus maze, EPM) and depression-like (tail suspension, TST, and forced swim, FST) behaviors. The ABX compounds were applied in drinking water during 7 days and consisted of a mixture of ampicillin (1mg/ml), streptomycin (5mg/ml), colistin (1mg/ml), vancomycin (0.5mg/ml) and amphotericin (0.1 mg/ml).

Supplementary Figure 5.

(A) Timeline of behavioral experiments using a separate cohort of sham- and Vx-operated animals. At 9 weeks following Vx or sham surgery, behavioral responses were assessed using a variety of standard tests for anxiety and depression**. (B)** Graph

depicting the total time spent in the center of the open field (OF), a trait of anxiety-related responses. (**C)** Total ambulatory activity in the open field during 20 minutes**.** **(D)** Total time spent in the open arms of the elevated plus maze (EPM), a readout of anxiety-related behavior. (**E)** Total time spent in the brightly-lit compartment of the light/dark box. **(F)** Graph showing the latency to eat a pellet following a 18 H fast in the novelty suppressed feeding test (NSF). **(G)** Feeding drive was assessed for 5 minutes following the end of the novelty suppressed feeding test and did not reveal any differences. **(H)** Total immobility in the tail suspension test (TST), a readout of depressive-like behaviors. **(I)** Total immobility in the forced swim test (FST). **(J)** Western blot membranes and their respective bar-plots reflecting the lack of difference in the effects of CT- and UCMS-derived gut microbiota on neurogenic factors in the hippocampus of vagotomized (Vx) mice. For western blot analysis, n=5/group were used. Data were analyzed using the non-parametric Mann-Whitney test.
