# Supplementary figures and images for: Gut microbiota changes require vagus nerve integrity to promote depressive-like behaviors in mice
Source: Mol Psychiatry. 2023 May 2;28(7):3002–12. doi: 10.1038/s41380-023-02071-6 (PMC10615761; doi:10.1038/s41380-023-02071-6)

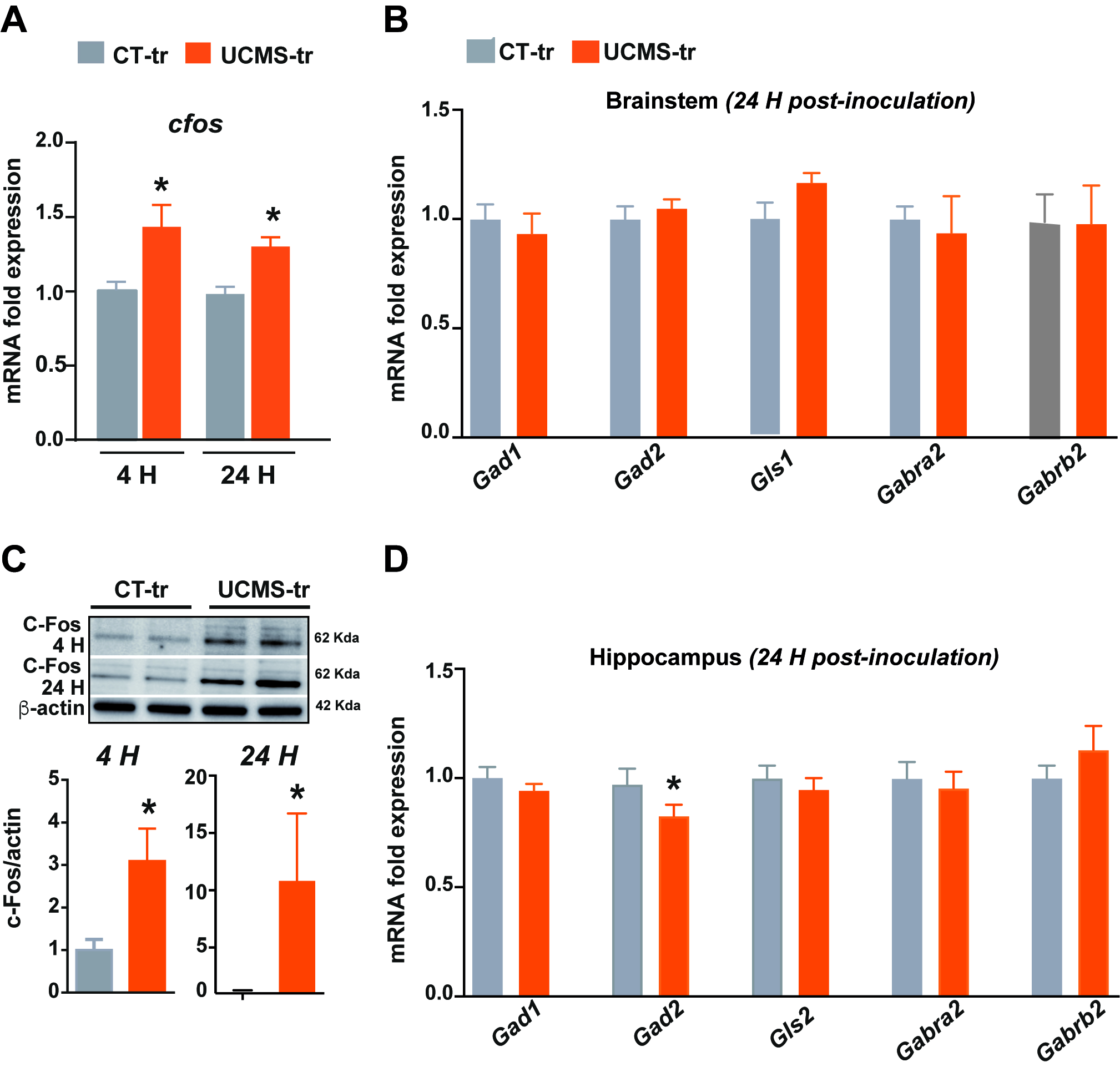

Supplement: Supplementary file 2 — Supplemental Figure 1 [file 41380_2023_2071_MOESM2_ESM.tif]

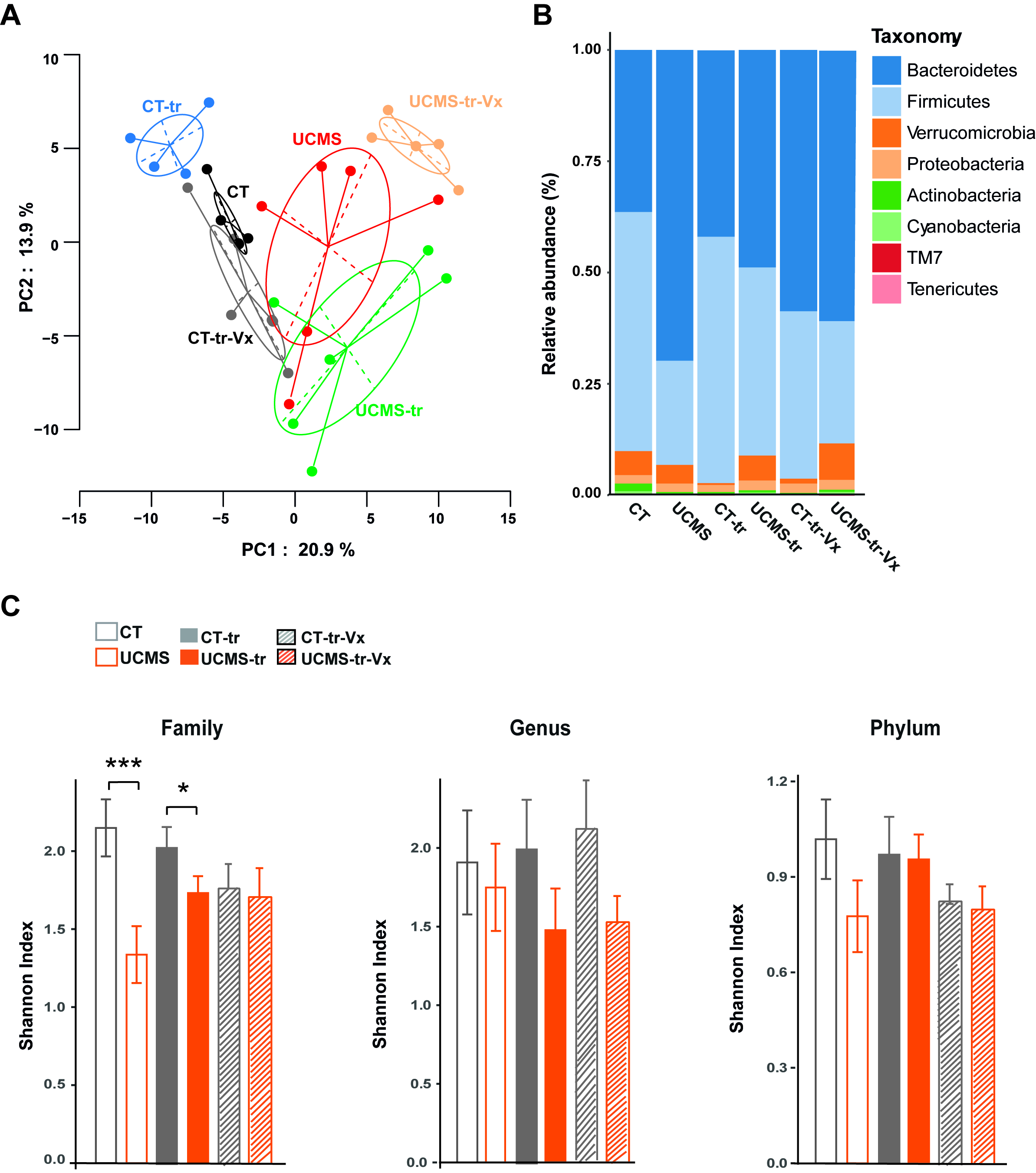

Supplement: Supplementary file 3 — Supplemental Figure 2 [file 41380_2023_2071_MOESM3_ESM.tif]

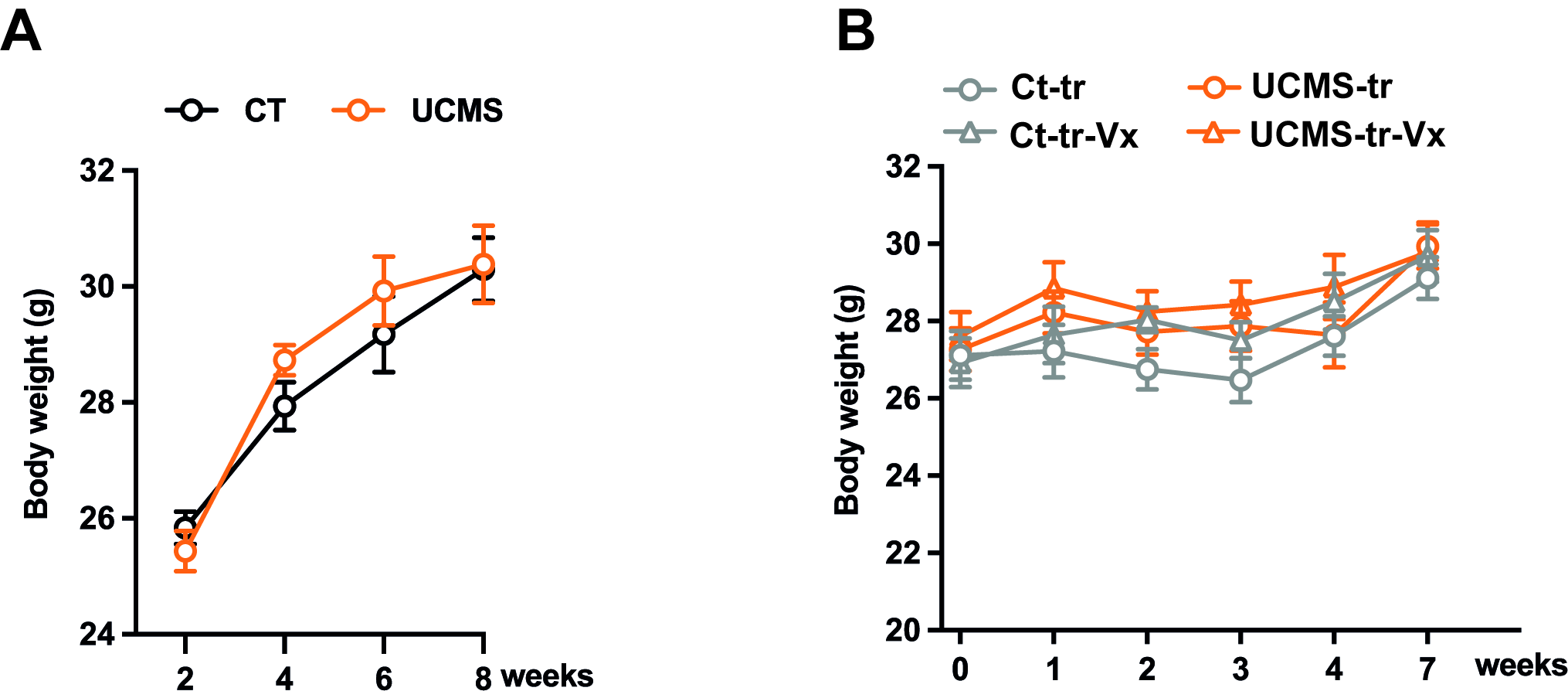

Supplement: Supplementary file 4 — Supplemental Figure 3 [file 41380_2023_2071_MOESM4_ESM.tif]

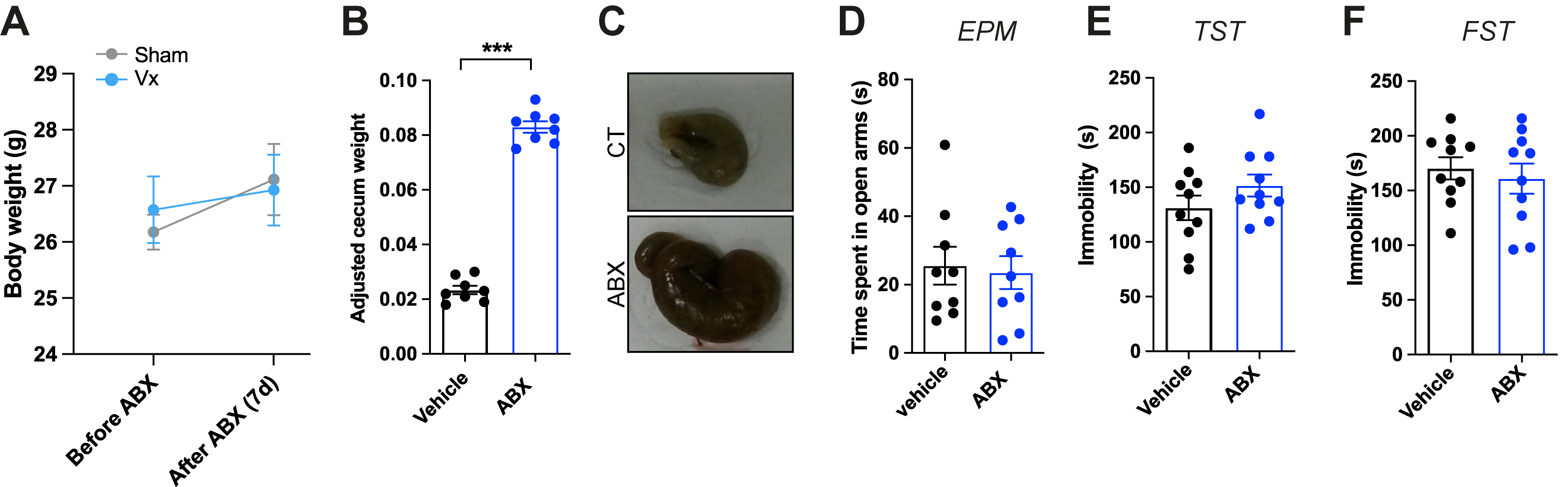

Supplement: Supplementary file 5 — Supplemental Figure 4 [file 41380_2023_2071_MOESM5_ESM.tif]

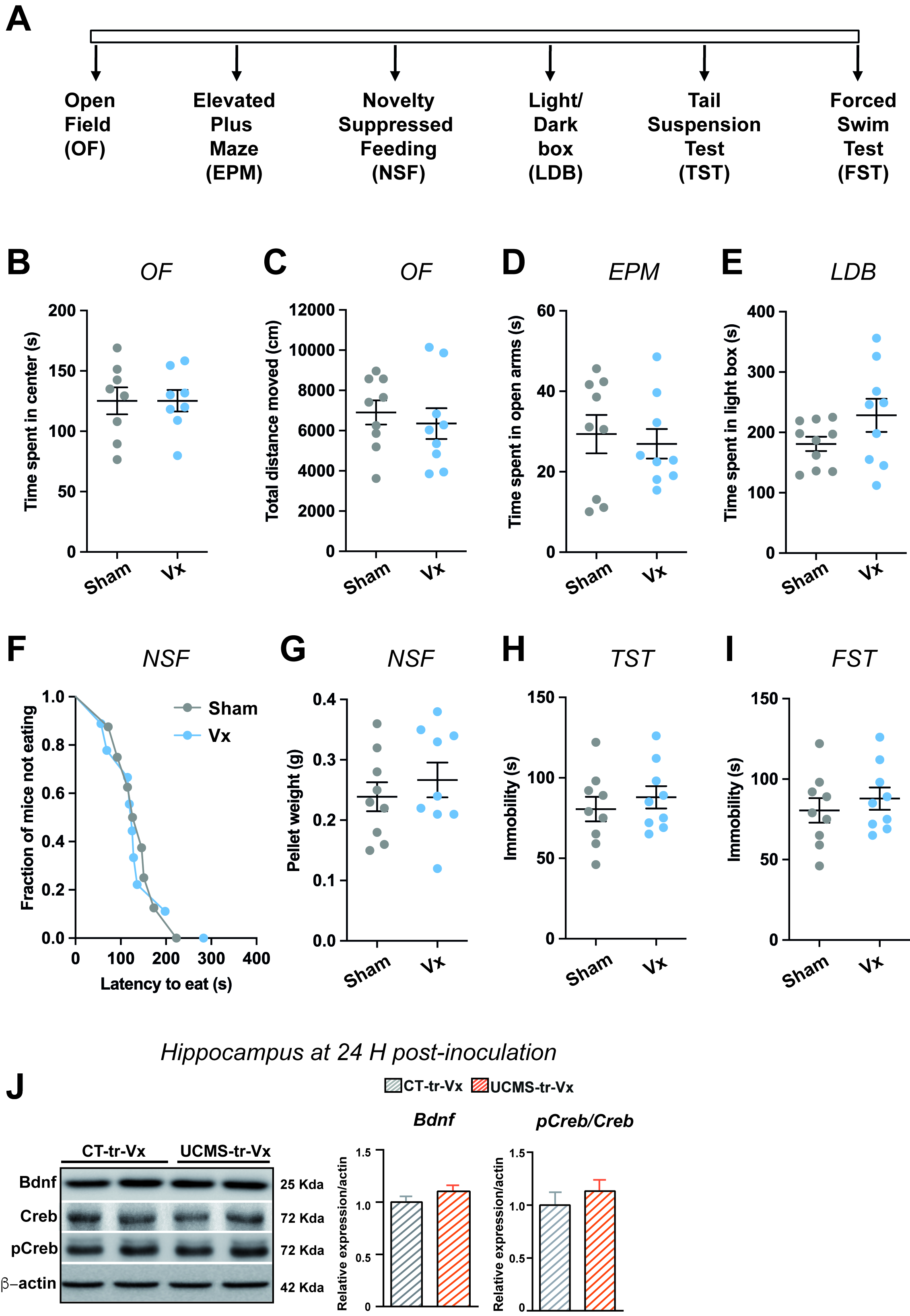

Supplement: Supplementary file 6 — Supplemental Figure 5 [file 41380_2023_2071_MOESM6_ESM.tif]

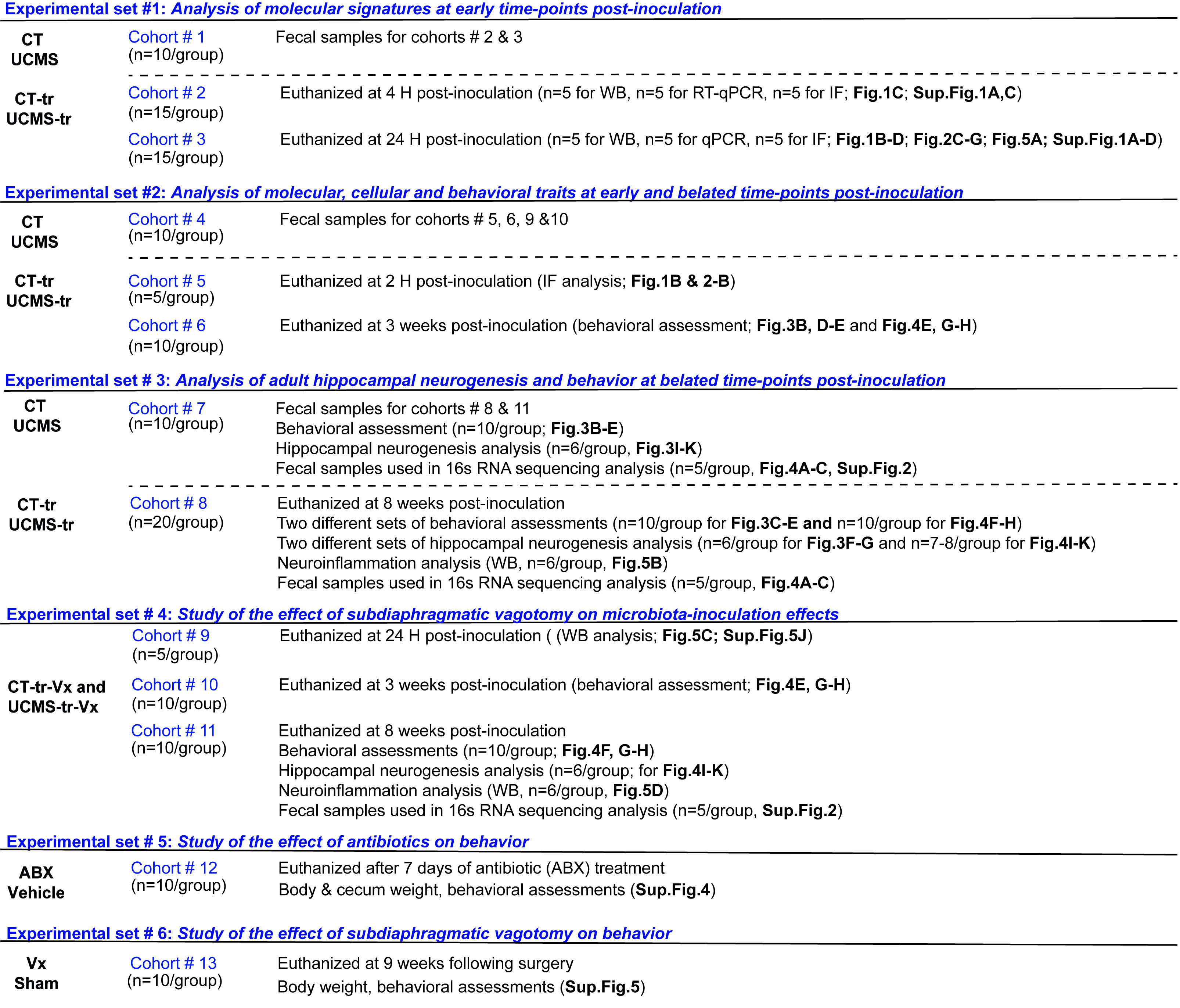

Supplement: Supplementary file 7 — Supplemental Table [file 41380_2023_2071_MOESM7_ESM.tif]
